# Severe back pain and neck/shoulder pain in experienced nurses in Sweden – a descriptive cross-sectional study of general health and pain characteristics, use of health resources and impact of pain on work

**DOI:** 10.1186/s12912-026-04542-x

**Published:** 2026-03-21

**Authors:** Eva Skillgate, Tobias Sundberg, Petter Gustavsson, Ann Rudman

**Affiliations:** 1 Musculoskeletal and Sports Injury Epidemiology Center, Department of Health Promotion Science, Sophiahemmet University, Valhallavägen 91, Box 5605, Stockholm, 114 86 Sweden; 2 Institute of Environmental Medicine, Karolinska Institutet, Stockholm, Sweden; 3 Department of Clinical Neuroscience, Division of Psychology, Karolinska Institutet, Stockholm, Sweden; 4 School of Health and Welfare, Department of Caring Sciences, Dalarna University, Stockholm, Sweden

**Keywords:** Back pain, Neck/shoulder pain, Nurses, Cohort study, Prevalence

## Abstract

**Background:**

The prevalence of back pain and neck/shoulder pain among nurses is high. This study reports a detailed description of health related characteristics of experienced nurses with and without severe back pain or severe neck/shoulder pain in Sweden.

**Methods:**

Cross-sectional data was collected in a national investigation of nursing graduates from 26 Swedish universities to identify three groups of nurses 11–15 years after graduation: nurses with severe back pain (*n* = 212), nurses with severe neck/shoulder pain (*n* = 277), and nurses with no or little such pain (*n* = 1525). Severe pain was defined as much discomfort from pain the preceding four weeks. Descriptive statistics were used to summarise general health and pain characteristics, use of health resources and impact of pain on work.

**Results:**

Nurses with severe back or neck/shoulder pain described worse general state of health and lifestyle, such as personal or mental health problems, fatigue, dizziness and pain in other body regions, less physical activity and lower sleep quality, than those with no or little pain. They used more health resources as over-the-counter and prescription painkillers and care seeking for physical ailments. The course of the severe pain was often persistent, and those nurses commonly reduced their working hours, changed work tasks, and had days off work.

**Conclusions:**

This study describes variations of nurses’ health related characteristics by pain levels. Experienced nurses with severe back or neck/shoulder pain describe worse states of general health and lifestyle, and more use of health resources than experienced nurses with no or little such pain. The course of the severe pain is often persistent and affects work capacity.

**Clinical trial number:**

Not applicable.

**Supplementary Information:**

The online version contains supplementary material available at 10.1186/s12912-026-04542-x.

## Background

Spinal pain including back pain and neck pain are frequent and economically burdensome musculoskeletal disorders and leading causes of disability [1–6]. Notably, the nursing profession is a vocation where disorders of the musculoskeletal system are common and back pain and neck/shoulder pain are highly prevalent [7–13]. Specifically, the nursing profession has a high prevalence of persistent or recurrent musculoskeletal disorders at all body regions, and there is a substantial prevalence of work-disabling shoulder pain and work-disabling and functional task-disabling back pain [11]. In Sweden, previous research of nurses has reported high and persisting prevalence of neck/shoulder pain (50%) and back pain (40%) among nursing students and early career nurses during their first two years after graduation [14].

Previous research involving nurses and their reported prevalence of pain in the back, neck and shoulder suggests that physical work tasks are associated with both back pain and shoulder pain, and that job strain has a strong association with neck pain [10]. Associations have also been reported between high psychosocial demands-low job control and prevalent shoulder pain and prevalent and incident back pain, and the latter disorder has also been associated with low social support [15]. Occupational psychosocial factors including high demands may also be of importance for nursing staff considering associations between such exposure and neck pain or discomfort in this occupational group [16].

Despite extensive work force policies and regulations, including both physical and ergonomic aspects [17] as well as organisational and psychosocial concerns [18], and despite recent advances in the knowledge of prevalence and associations of importance for back pain and neck/shoulder pain in nurses, there is currently a lack of knowledge and research informed consensus regarding which interventions or strategies that are best to use in order to prevent or rehabilitate back pain, or decrease the frequency and impact of musculoskeletal pain, in nurses [19–21]. Nonetheless, it has been suggested that several factors associated with back pain and neck/shoulder pain in nurses might be modifiable, such as less physical activity, physical work load, overtime work and sickness presence, and thus may be targeted by preventive strategies [14]. However, there is a research gap in the prevalence of such characteristics among experienced nurses and across pain levels. A deeper understanding of health related characteristics and the back pain and the neck/shoulder pain that nurses experience may contribute toward informing future studies investigating effective prevention and rehabilitation including work retention strategies among experienced nurses with severe pain.

### Aim and objectives

The aim of this study was to report a detailed description of health related characteristics of experienced nurses with and without severe back pain or severe neck/shoulder pain in Sweden. Specifically, the objective was to describe and characterize the health profiles of experienced nurses to identify factors associated with severe back pain and neck/shoulder pain. By analyzing the impact of these conditions on health, work and resource use 11–15 years after graduation, this study may contribute essential evidence for informing organizational strategies aimed at reducing work-related disability and identify critical target areas for intervention strategies sustaining a senior nursing workforce.

## Methods

### Data collection, design and setting

**Fig. 1:**
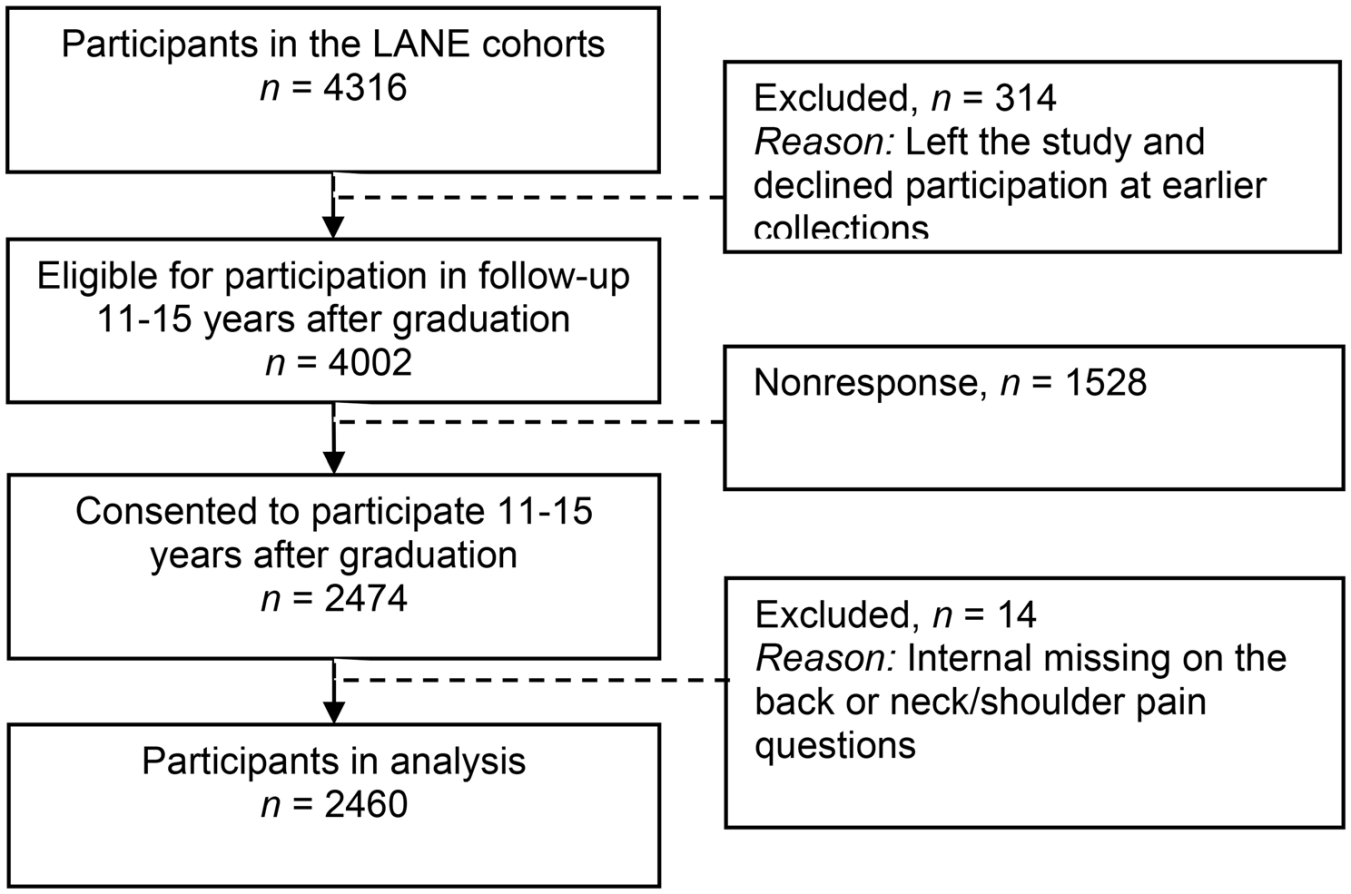
Recruitment and participation flowchart for the three cohorts included in the LANE study 11–15 years after graduation

Cross-sectional primary data was collected from licensed nurses 11–15 years after their graduation in the Longitudinal Analysis of Nursing Education (LANE) study, a long term national investigation of nursing graduates from 26 Swedish universities in 2002, 2004 and 2006 [22]. These cohorts were prospectively followed through 2010 [22]. In 2017, a long-term follow‑up 11–15 years after graduation was initiated, including the 4,002 individuals from the original cohorts who were still eligible to participate [23]. The methodological basis for the LANE study has been detailed in previously published research [22, 23]. The recruitment and participation of nurses in the LANE study is presented in a flowchart (Fig. 1). There were originally 4316 participants in the LANE study, of which 4002 were eligible and 2474 consented to participate in the longer term data collection 11–15 years after graduation (Fig. 1). In this sample, 2460 nurses with information about back pain or neck/shoulder pain were identified and included for participation in the current study by their responses to a check list of health problems entitled “Have you had any of the following health problems in the last 4 weeks?“. The symptoms of back pain and neck/shoulder pain were self-rated on a scale with four response categories: none (no), mild (little discomfort), moderate (yes, moderate discomfort), and severe (yes, much discomfort). To explore and emphasize nurses with high pain experiences in the current study, only observations of nurses that were categorised into the extremes of pain intensity were included. Thus the employed inclusion criteria were observations of nurses rating severe back pain (n = 212) and severe neck/shoulder pain (n = 277) respectively, and observations of nurses that rated their pain as “none” or “mild” (n = 1525), which were categorised as having “no or little” back or neck/shoulder pain. Observations of nurses rating their pain as “moderate” were not included. Statistics Sweden provided technical assistance and managed the survey logistics and data collection, this support included pilot-testing of all questions and reviewing of each survey by staff at Statistics Sweden’s technical and language laboratory [24].

### Measurements

The surveys contained items relating to the prevalence and severity of back pain and neck/shoulder pain, demographic characteristics and a range of background and work-related variables, which have been described in detail in previously published research [14, 22]. In short, survey items were derived from a mix of questions from established questionnaires and study-specific questions developed by consensus in the research group. The questions were then categorised into the following three domains.

1. Questions related to demographic and general health characteristics. These questions capture participants’ demographic profile and overall health status, including age, sex, and occupational category, alongside self-reported general health and lifestyle factors. They also assess recent symptoms and health behaviors such as physical activity, smoking, sleep quality, fatigue, and dizziness within the past four weeks.
2. Questions related to pain characteristics. These questions assess the presence, anatomical distribution, and temporal course of musculoskeletal pain across multiple body regions, including the spine and extremities. They capture both recent pain (past 4 weeks) and longer-term pain trajectories over the past 12 months, providing an overview of pain prevalence and persistence.
3. Questions related to use of health resources and impact of pain on work characteristics. These questions evaluate healthcare utilization and medication use related to physical and mental health, as well as the occupational impact of back and neck problems. They assess pain-related work impairment, including reduced performance, task modification, decreased working hours, and sickness absence.

Supplement 1 reports the specific questions and response categories used to provide a detailed description of the characteristics of the nurses with relevance to demographic, health, pain, health resource use and work related aspects of back pain and neck/shoulder pain.

### Data analysis

The SPSS 25 and STATA 16 statistical software packages were used to analyze survey data and produce outputs of descriptive statistics to present a detailed description of the nurses. Categorical data were reported as frequency distributions and percentages with 95% confidence intervals to indicate the precision of estimates using standard binomial methods. Nurses with severe back pain, and severe neck/shoulder pain, were described in relation to each other and to nurses with no or little back pain or neck/shoulder pain.

## Results

There were 2460 experienced nurses that participated in the longer term follow up 11–15 years after graduation with information about back pain or neck/shoulder pain (Fig. 1). Of these, 212 (9%) nurses reported severe back pain, 277 (11%) reported severe neck/shoulder pain, and 1525 (62%) reported no back or neck/shoulder pain.

The detailed description of the characteristics of the nurses are presented in three tables, one for each domain: Demographic and general health characteristics (Table 1), pain characteristics (Table 2), and the use of health resources and work related characteristics (Table 3).

**Table 1:** Demographic and general health characteristics of experienced nurses with severe back pain, severe neck/shoulder pain, and no or little back or neck/shoulder pain, 11–15 years after graduation

		Severe back pain		Severe neck/shoulder pain		No or little back pain or neck/shoulder pain	
		*N* = 212		*N* = 277		*N* = 1 525	
Characteristic	Category	N	Percent (95% CI)	N	Percent (95% CI)	N	Percent (95% CI)
Sex	Woman	200	94% (90% to 97%)	269	97% (94% to 99%)	1336	88% (86% to 89%)
	Man	12	6% (3% to 10%)	8	3% (1% to 6%)	189	12% (11% to 14%)
Age (years)	≤ 39	72	34% (28% to 41%)	108	39% (33% to 45%)	647	42% (40% to 45%)
	40–49	75	35% (29% to 42%)	100	36% (30% to 42%)	520	34% (32% to 37%)
	≥ 50	65	31% (25% to 37%)	69	25% (20% to 30%)	358	23% (21% to 26%)
Occupational category	Registered nurse	92	43% (37% to 50%)	114	41% (35% to 47%)	533	35% (33% to 37%)
	Specialist nurse	82	39% (32% to 46%)	108	39% (33% to 45%)	691	45% (43% to 48%)
	Midwife	13	6% (3% to 10%)	22	8% (5% to 12%)	71	5% (4% to 6%)
	Other	6	3% (1% to 6%)	9	3% (1% to 6%)	41	3% (2% to 4%)
Self-rated general state of health	Good	37	17% (13% to 23%)	44	16% (12% to 21%)	840	55% (53% to 58%)
	Fairly good	79	37% (31% to 44%)	118	43% (37% to 49%)	551	36% (34% to 39%)
	Neither good nor bad	58	27% (21% to 34%	68	25% (20% to 30%)	96	6% (5% to 8%)
	Fairly bad	34	16% (11% to 22%)	37	13% (10% to 18%)	27	2% (1% to 3%)
	Bad	4	2% (1% to 5%)	9	3% (1% to 6%)	5	0% (0% to 1%)
Physical activity by exercise or sports	Daily	8	4% (2% to 7%)	14	5% (3% to 8%)	91	6% (5% to 7%)
	Several times a week	58	27% (21% to 34%)	67	24% (19% to 30%)	605	40% (37% to 42%)
	Sometime a week	55	26% (20% to 32%)	72	26% (21% to 32%)	406	27% (24% to 29%)
	Sometime/a few times a month	44	21% (16% to 27%)	63	23% (18% to 28%)	231	15% (13% to 17%)
	Never	47	22% (17% to 28%)	61	22% (17% to 27%)	189	12% (11% to 14%)
Do you smoke?	Yes, regularly	14	7% (4% to 11%)	19	7% (4% to 11%)	39	3% (2% to 3%)
	Yes, on occasion	9	4% (2% to 8%)	13	5% (3% to 8%)	67	4% (3% to 6%)
	No	188	89% (84% to 93%)	244	88% (84% to 92%)	1419	93% (92% to 94%)
How do you assess your sleep quality?	Good	27	13% (9% to 18%)	34	12% (9% to 17%)	482	32% (29% to 34%)
	Fairly good	49	23% (18% to 29%)	71	26% (21% to 31%)	636	42% (39% to 44%)
	Neither good nor bad	59	28% (22% to 34%)	81	29% (24% to 35%)	266	17% (16% to 19%)
	Fairly bad	58	27% (21% to 34%)	67	24% (19% to 30%)	125	8% (7% to 10%)
	Bad	19	9% (5% to 14%)	23	8% (5% to 12%)	14	1% (1% to 2%)
Have you had fatigue in the last 4 weeks?	No	13	6% (3% to 10%)	11	4% (2% to 7%)	345	23% (21% to 25%)
	Yes, mild	41	19% (14% to 25%)	65	23% (19% to 29%)	769	50% (48% to 53%)
	Yes, moderate	87	41% (34% to 48%)	97	35% (29% to 41%)	317	21% (19% to 23%)
	Yes, severe	71	33% (27% to 40%)	102	37% (31% to 43%)	89	6% (5% to 7%)
Have you had dizziness in the last 4 weeks?	No	108	51% (44% to 58%)	124	45% (39% to 51%)	1187	78% (76% to 80%)
	Yes, mild	68	32% (26% to 39%)	102	37% (31% to 43%)	274	18% (16% to 20%)
	Yes, moderate	28	13% (9% to 19%)	35	13% (9% to 17%)	52	3% (3% to 4%)
	Yes, severe	8	4% (2% to 7%)	16	6% (3% to 9%)	5	0% (0% to 1%)

CI, Confidence interval

**Table 2:** Pain characteristics of experienced nurses with severe back pain, severe neck/shoulder pain, and no or little back or neck/shoulder pain, 11–15 years after graduation

		Severe back pain		Severe neck/shoulder pain		No or little back pain or neck/shoulder pain	
		*N* = 212		*N* = 277		*N* = 1 525	
Characteristic	Category	N	Percent (95% CI)	N	Percent (95% CI)	N	Percent (95% CI)
Have you had back pain in the last 4 weeks?	No	-	-	53	19% (15% to 24%)	957	63% (60% to 65%)
	Yes, mild	-	-	44	16% (12% to 21%)	568	37% (35% to 40%)
	Yes, moderate	-	-	68	25% (20% to 30%)	-	-
	Yes, severe	212	100% (98% to 100%)	112	40% (35% to 46%)	-	-
Have you had neck/shoulder pain in the last 4 weeks?	No	30	14% (10% to 20%)	-	-	814	53% (51% to 56%)
	Yes, mild	22	10% (7% to 15%)	-	-	711	47% (44% to 49%)
	Yes, moderate	46	22% (16% to 28%)	-	-	-	-
	Yes, severe	112	53% (46% to 60%)	277	100% (99% to 100%)	-	-
Course of pain in the lower back in the last 12 months?	Have not had pain in the lower back	9	4% (2% to 8%)	69	25% (20% to 30%)	-	-
	A single occasion with pain and mainly pain-free before that	8	4% (2% to 7%)	24	9% (6% to 13%)	-	-
	A few occasions with pain, with mainly pain-free periods in between	29	14% (9% to 19%)	68	25% (20% to 30%)	-	-
	Slight but noticeable pain most of the time and a couple of times with severe pain	58	27% (21% to 34%)	46	17% (12% to 22%)	-	-
	Pain that goes up and down all the time, with occasions of severe pain	71	33% (27% to 40%)	51	18% (14% to 23%)	-	-
	Pain all or almost all the time	35	17% (12% to 22%)	16	6% (3% to 9%)	-	-
Course of upper back pain in the last 12 months?	Have not had pain in the upper back	30	14% (10% to 20%)	8	3% (1% to 6%)	-	-
	A single occasion with pain and mainly pain-free before that	18	8% (5% to 13%)	8	3% (1% to 6%)	-	-
	A few occasions with pain, with mainly pain-free periods in between	59	28% (22% to 34%)	52	19% (14% to 24%)	-	-
	Slight but noticeable pain most of the time and a couple of times with severe pain	57	27% (21% to 33%)	105	38% (32% to 44%)	-	-
	Pain that goes up and down all the time, with occasions of severe pain	28	13% (9% to 19%)	68	25% (20% to 30%)	-	-
	Pain all or almost all the time	18	8% (5% to 13%)	35	13% (9% to 17%)	-	-
Have you had a headache in the last 4 weeks?	No	36	17% (12% to 23%)	31	11% (8% to 16%)	533	35% (33% to 37%)
	Yes, mild	72	34% (28% to 41%)	73	26% (21% to 32%)	726	48% (45% to 50%)
	Yes, moderate	63	30% (24% to 36%)	93	34% (28% to 39%)	211	14% (12% to 16%)
	Yes, severe	40	19% (14% to 25%)	79	29% (23% to 34%)	49	3% (2% to 4%)
Have you had pain in the jaw joints in the last 4 weeks?	No	134	63% (56% to 70%)	149	54% (48% to 60%)	1355	89% (87% to 90%)
	Yes, mild	41	19% (14% to 25%)	57	21% (16% to 26%)	140	9% (8% to 11%)
	Yes, moderate	11	5% (3% to 9%)	28	10% (7% to 14%)	18	1% (1% to 2%)
	Yes, severe	25	12% (8% to 17%)	41	15% (11% to 20%)	8	1% (0% to 1%)
Have you had pain in your arms and/or hands in the last 4 weeks?	No	90	42% (36% to 49%)	98	35% (30% to 41%)	1241	81% (79% to 83%)
	Yes, mild	37	17% (13% to 23%)	45	16% (12% to 21%)	236	15% (14% to 17%)
	Yes, moderate	36	17% (12% to 23%)	57	21% (16% to 26%)	41	3% (2% to 4%)
	Yes, severe	47	22% (17% to 28%)	74	27% (22% to 32%)	7	0% (0% to 1%)
Have you had hip pain in the last 4 weeks?	No	77	36% (30% to 43%)	133	48% (42% to 54%)	1245	82% (80% to 84%)
	Yes, mild	44	21% (16% to 27%)	51	18% (14% to 23%)	225	15% (13% to 17%)
	Yes, moderate	39	18% (13% to 24%)	42	15% (11% to 20%)	40	3% (2% to 4%)
	Yes, severe	51	24% (18% to 30%)	51	18% (14% to 23%)	11	1% (0% to 1%)
Have you had knee pain in the last 4 weeks?	No	110	52% (45% to 59%)	162	58% (52% to 64%)	1252	82% (80% to 84%)
	Yes, mild	40	19% (14% to 25%)	49	18% (13% to 23%)	200	13% (11% to 15%)
	Yes, moderate	34	16% (11% to 22%)	40	14% (11% to 19%)	57	4% (3% to 5%)
	Yes, severe	26	12% (8% to 17%)	26	9% (6% to 13%)	15	1% (1% to 2%)
Have you had pain in your ankles in the last 4 weeks?	No	155	73% (67% to 79%)	206	74% (69% to 79%)	1447	95% (94% to 96%)
	Yes, mild	19	9% (5% to 14%)	29	10% (7% to 15%)	54	4% (3% to 5%)
	Yes, moderate	17	8% (5% to 13%)	24	9% 6% to 13%)	14	1% (1% to 2%)
	Yes, severe	18	8% (5% to 13%)	17	6% (4% to 10%)	6	0% (0% to 1%)
Have you had pain in your feet in the last 4 weeks?	No	102	48% (41% to 55%)	151	55% (48% to 60%)	1242	81% (79% to 83%)
	Yes, mild	43	20% (15% to 26%)	54	19% (15% to 25%)	191	13% (11% to 14%)
	Yes, moderate	29	14% (9% to 19%)	35	13% (9% to 17%)	69	5% (4% to 6%)
	Yes, severe	36	17% (12% to 23%)	37	13% (10% to 18%)	22	1% (1% to 2%)

CI, Confidence interval

**Table 3:** Use of health resources and work related characteristics of experienced nurses with severe back pain, severe neck/shoulder pain, and no or little back or neck/shoulder pain, 11–15 years after graduation

		Severe back pain		Severe neck/shoulder pain		No or little back pain or neck/shoulder pain	
		*N* = 212		*N* = 277		*N* = 1 525	
Characteristic	Category	N	Percent (95% CI)	N	Percent (95% CI)	N	Percent (95% CI)
How often have you used over-the-counter painkillers in the last 4 weeks	Virtually every day	26	12% (8% to 17%)	26	9% (6% to 13%)	29	2% (1% to 3%)
	Several times a week	51	24% (18% to 30%)	72	26% (21% to 32%)	76	5% (4% to 6%)
	Sometime a week	53	25% (19% to 31%)	78	28% (23% to 34%)	224	15% (13% to 17%)
	Every once in a while	51	24% (18% to 30%)	62	22% (18% to 28%)	781	51% (49% to 54%)
	Never	24	11% (7% to 16%)	31	11% (8% to 16%)	407	27% (24% to 29%)
How often have you used prescription painkillers in the last 4 weeks	Virtually every day	27	13% (9% to 18%)	28	10% (7% to 14%)	19	1% (1% to 2%)
	Several times a week	10	5% (2% to 9%)	12	4% (2% to 7%)	4	0% (0% to 1%)
	Sometime a week	10	5% (2% to 9%)	12	4% (2% to 7%)	17	1% (1% to 2%)
	Every once in a while	26	12% (8% to 17%)	26	9% (6% to 13%)	56	4% (3% to 5%)
	Never	135	64% (57% to 70%)	192	69% (64% to 75%)	1406	92% (91% to 93%)
Have you sought care for physical ailments in the last 12 months?	Yes, in the last 3 months	102	48% (41% to 55%)	123	44% (38% to 50%)	263	17% (15% to 19%)
	Yes, earlier in the year - but not in the last 3 months	48	23% (17% to 29%)	61	22% (17% to 27%)	296	19% (17% to 21%)
	No	62	29% (23% to 36%)	93	34% (28% to 39%)	957	63% (60% to 65%)
Have you sought care for personal problems or mental health problems in the last 12 months?	Yes, in the last 3 months	39	18% (13% to 24%)	54	19% (15% to 25%)	86	6% (5% to 7%)
	Yes, earlier in the year - but not in the last 3 months	35	17% (12% to 22%)	48	17% (13% to 22%)	124	8% (7% to 10%)
	No	137	65% (58% to 71%)	174	63% (57% to 69%)	1308	86% (84% to 87%)
To what extent have you experienced back/neck problems affecting work performance?	Not at all	28	13% (9% to 19%)	43	16% (11% to 20%)	-	-
	To a small degree	58	27% (21% to 34%)	94	34% (28% to 40%)	-	-
	To a moderate degree	54	25% (20% to 32%)	80	29% (24% to 35%)	-	-
	To a high degree	36	17% (12% to 23%)	34	12% (9% to 17%)	-	-
	Have not worked	6	3% (1% to 6%)	9	3% (1% to 6%)	-	-
To what extent have you reduced working hours/changed work tasks due to back/neck problems?	Not at all	122	58% (51% to 64%)	199	72% (66% to 77%)	-	-
	To a small degree	21	10% (6% to 15%)	19	7% (4% to 11%)	-	-
	To a moderate degree	13	6% (3% to 10%)	20	7% (4% to 11%)	-	-
	To a high degree	16	8% (4% to 12%)	13	5% (3% to 8%)	-	-
	Have not worked	10	5% (2% to 9%)	11	4% (2% to 7%)	-	-
How many days have you been away from work due to back/neck problems?	0	134	63% (56% to 70%)	215	78% (72% to 82%)	-	-
	< 25	34	16% (11% to 22%)	32	12% (8% to 16%)	-	-
	> 50	9	4% (2% to 8%)	6	2% (1% to 5%)	-	-

CI, Confidence interval

The vast majority of the participants were women and occupied as registered nurses with or without specialist training. Those with severe back pain and those with severe neck/shoulder pain shared several characteristics compared to those with no or little such pains; they described their general state of health worse, participated less in physical activity, had lower sleep quality, and had more problems with fatigue and dizziness and pain in other body regions. The nurses with severe back pain and severe neck/shoulder pain also used more over-the-counter and prescription pain killers in the last month, and sought more care for physical ailments and personal or mental health problems in the last year, compared to the nurses with no or little back pain or neck/shoulder pain. The majority of nurses with severe back pain and severe neck/shoulder pain described that their back/neck problems affected their work capacity to at least some degree. There were more nurses with severe back pain than nurses with severe neck/shoulder pain that described they had reduced their working hours or changed work tasks, and had days away from work, due to their back/neck problems.

## Discussion

The aim of this study was to deepen the knowledge about severe back pain and severe neck/shoulder pain in experienced nurses by the description of demographic and general health characteristics, pain related characteristics, and the use of health resources and impact of pain on work characteristics.

### Demographic and health related characteristics

The majority of all participating nurses were women, although there were somewhat more men in the group with no or little back pain or neck/shoulder pain compared to the other two groups. There were comparable distributions of participants over the different age categories across groups. Results from global study data have shown that both age-standardized prevalence of back pain, and years lived with disability, is higher among women than men [6].

Most participants were working as nurses or specialist nurses. It is known from previous research that this occupational group has a high prevalence of musculoskeletal disorders [7–12]. The current study adds to this knowledge by showing that nurses with severe back pain or severe neck/shoulder pain mostly rated their general state of health as “neither good nor bad” or “fairly good”, whereas the majority of those with no or little back pain or neck/shoulder pain mostly rated their general health in the highest category “good”. This is in line with previous research reporting associations of more back pain or neck pain and more mental health problems including depression and anxiety [28].

There were nearly twice as many nurses with severe back pain or severe neck/shoulder pain (22%) that never participated in physical activity by exercise or sports compared to the nurses with no or little back pain or neck/shoulder pain (12%). Conversely there were more nurses in the latter group that participated in physical activity several times a week (40%) compared to those with pain (24–27%) (Table 1). A study from Norway on university students reported an association between higher participation in physical activities and lesser pain, and that participation in activities thus should be encouraged [29]. The current study findings further showed that regular smoking was more than twice as common in nurses with severe back pain or severe neck/shoulder pain compared to those with no or little such pain. However, in a recent systematic review there was inconsistent association for smoking as a risk factor for back pain, whereas there was moderate quality evidence that previous back pain was a risk factor for back pain [30].

Current research suggests there is weak to moderate evidence that more severe spinal pain may be associated with more disturbed sleep [31]. Our findings agree to that and showed that nurses with severe back pain or severe neck/shoulder pain report worse sleeping quality, fatigue and dizziness compared to nurses with no or little such pain (Table 1), suggesting an interrelatedness between these symptoms.

### Pain related characteristics

Over half (53%) of the nurses with severe back pain also reported severe neck/shoulder pain, whereas fewer (40%) among those with severe neck/shoulder pain also reported severe back pain, over the last four weeks. Additionally, severe musculoskeletal pains were also reported in other body regions, including e.g. headaches and upper and lower extremity pains, by up to 24% of nurses with severe back pain, and by up to 29% of nurses with severe neck/shoulder pain. This shows that comorbidity of musculoskeletal disorders among the participating nurses was common, a phenomenon that concur with previous studies [7–8, 10–12].

About one fifth (17%) of the nurses with severe back pain had lower back pain all or almost all the time, while the most frequently (33%) reported course of low back pain was pain that constantly fluctuated with occasions of severe pain. Conversely, almost as many (13%) of the nurses with severe neck/shoulder pain had upper spinal pain all or almost all the time, while the most reported course (38%) was slight but notable pain most of the time and a couple of times with severe pain. Notably, only 4% of the nurses with severe back pain, and only 3% of the nurses with severe neck/shoulder pain, reported no pain in the lower back or upper back over the last four weeks respectively. This indicates that frequent pain is common as is the co-occurance of pain across body regions, thus that continuous or pain management strategies including self-management may be of importance to the participating nurses.

### Use of health resources and work related characteristics

The use of pharmacological pain killers, both over-the-counter and prescription based, were common pain management strategies among the responding nurses. The majority of nurses in each pain cohort reported using over-the-counter pain killers at least sometime a week to every day. Among the nurses with severe back pain, there were approximately just as many that used over-the-counter pain killers every day (12%) that were also using prescription pain killers every day (13%). Among the nurses with severe neck/shoulder pain similar figures were reported of 9% and 10% respectively. Considering potential adverse events and uncertainty of safety and effectiveness of long-term use of common analgesics, including non-steroidal anti-inflammatory drugs, paracetamol and opioids, for musculoskeletal back pain, it may not be generally recommended to use such analgesics for extended lengths of times [32–34]. The use of non-pharmacological pain management strategies for nurses may be considered. A Swedish study investigated health promotion activities for nurses and nurse assistants provided at work, as a complement to conventional care for staff, supporting that some on-site interventions (massage and/or hypnosis) may be valuable for some nurses to decrease pain and stress [35]. However, there is no compelling systematic research evidence of effective interventions to reduce or prevent work related pain in nurses [19, 21]. Thus, further research into proactive and health promotion activities for nurses and nursing students are needed, which may include technical devices and other work environment adjustments at the organizational level that support nurses in their occupational activities [36].

The vast majority of the nurses with severe back pain or severe neck/shoulder pain reported having sought care for physical ailments (71% and 66% respectively), and to a lesser extent having sought care for personal or mental health problems (35% and 37% respectively), in the last year. The corresponding figures of care seeking in the last year in the nurses with no or little back pain or neck/shoulder pain were approximately half, represented by 37% for physical ailments and 14% for personal or mental health problems. Although these figures do not report the numbers or types of care visits, they suggest that nurses with severe back pain or severe neck/shoulder pain are likely using care resources to a greater extent than those with lesser pain. Possibly, this may not only be a personal burden to the individual nurses but also affect the nurses employers. Interestingly, most (58–72%) of the nurses with severe back pain or neck/shoulder pain reported they had not reduced their working hours or changed work tasks, nor that they had any days away from work (63–78%), due to their back or neck/shoulder related problems. Seemingly, the relatively high use of prescription and non-prescription pain killers, is one way for nurses to self manage and care for their back or neck/shoulder pain at work. A relevant topic for future studies.

### Methodological considerations

The current study reports data from the LANE study, the largest longitudinal study of the nursing profession in Sweden to date, and the findings are likely representative of the Swedish nursing profession at large [22]. There are however limitations that need to be considered with interpreting the study findings. A potential threat to the validity of self-reported pain findings is the risk of self selection bias, i.e. that nurses with more pain would be more likely to respond to the survey, however the LANE study collected data on a wide range of characteristics and work-related factors not only pain. Another risk is that the methods used to measure back pain and neck/shoulder pain are not optimal, meaning that measurement errors might have resulted in wrongly estimated prevalence of pain. Recall problems is another risk found in survey investigations that may hinder valid reporting of pain and other self reported findings in the study. Categorising nurses into the extremes of pain intensity, no/mild vs. severe, was done to clarify differences that may otherwise be obscured by the heterogeneity of moderate pain classification. Although this descriptive approach highlights the contrast between those with minimal versus high pain it does so at the expense of not representing intermediate pain experiences, which may still have clinical and occupational significance and be considered in future studies informing preventive or rehabilitative strategies. The questions targeting pain were developed under the lead of author ES and agreed upon by research group consensus. Albeit apparent face validity of the pain questions by the research group there has been no specific psychometric testing of the pain questions establishing their validity and reliability, which is a limitation. Given that this study employed a descriptive, hypothesis-generating design, we report cohort proportions with corresponding 95% confidence intervals without conducting formal hypothesis testing, and the resulting estimates should therefore be interpreted as exploratory rather than confirmatory. This approach allows for a broad characterization of patterns within the data and supports the identification of potentially meaningful associations for future hypothesis-driven research.

## Conclusions

This study describes that severe back pain and severe neck/shoulder pain among experienced nurses (11–15 years post-graduation) are not merely isolated physical ailments, but are linked to a broader decline in general state of health, less participation in physical activity, lower sleep quality, and more problems with fatigue and dizziness and pain in other body areas. Concomitantly, health resource use including over-the-counter and prescription pain killers in the last month, and seeking care for physical ailments and personal or mental health problems in the last year, were also described as more common in nurses with severe back pain or severe neck/shoulder pain compared to those with no or little such pains. Further, our study suggests that severe back pain and neck/shoulder pain may impact the nursing workforce stability and work capacity, whereby more nurses with severe back pain than neck/shoulder pain reported they had reduced their working hours, changed work tasks, and had days away from work, due to their back/neck pain.

Taken together, these findings propose that “one-size-fits-all” ergonomic or health promotion strategies may be insufficient to improve health and work capacity among experienced nurses with severe back/neck pain. Instead, targeted multifaceted strategies integrating specialized pain management and flexible work redesign are presumably needed to support health, retention, and sustainable workforce participation.

## Supplementary Information

Below is the link to the electronic supplementary material.

Supplementary Material 1

## Data Availability

The datasets used and/or analysed during the current study will not be accessible to others due to ethical constraints of data confidentiality.
