# Supplementary material for: Severe back pain and neck/shoulder pain in experienced nurses in Sweden – a descriptive cross-sectional study of general health and pain characteristics, use of health resources and impact of pain on work
Source: BMC Nurs. 2026 Mar 21;25:335. doi: 10.1186/s12912-026-04542-x (PMC13063674; doi:10.1186/s12912-026-04542-x)
Supplement: Supplementary file 1 — Supplementary Material 1 [file 12912_2026_4542_MOESM1_ESM.docx]

**Supplement 1.** Questions and response categories used to provide a detailed description of the characteristics of the nurses with relevance to demographic, health, pain, health resource use and work related aspects of back pain and neck/shoulder pain.

1) Questions (response categories) related to demographic and general health characteristics: Sex (Woman; Man); Age (Years: ≤ 39; 40-49; ≥ 50); Occupational category (Registered nurse; Specialist nurse; Midwife; Other); Self-rated general state of health (25) (Good; Fairly good; Neither good nor bad; Fairly bad; Bad); Physical activity by exercise or sports (Daily; Several times a week; Sometime a week; Sometime/a few times a month; Never); Do you smoke? (Yes, regularly; Yes, on occasion; No); How do you assess your sleep quality? (26) (Good; Fairly good; Neither good nor bad; Fairly bad; Bad); Have you had fatigue in the last 4 weeks? (No; Yes, mild; Yes, Moderate; Yes, severe); Have you had dizziness in the last 4 weeks? (No; Yes, mild; Yes, Moderate; Yes, severe).

2) Questions (response categories) related to pain characteristics:

Have you had back pain in the last 4 weeks? (No; Yes, mild; Yes, moderate; Yes, severe); Have you had neck/shoulder pain in the last 4 weeks? (No; Yes, mild; Yes, moderate; Yes, severe); Course of pain in the lower back in the last 12 months? (27) (Have not had pain in the lower back; A single occasion with pain and mainly pain-free before that; A few occasions with pain with mainly pain-free periods in between; Slight but noticeable pain most of the time and a couple of times with severe pain; Pain that goes up and down all the time with occasions of severe pain; Pain all or almost all the time); Course of upper back pain in the last 12 months? (27) (Have not had pain in the upper back; A single occasion with pain and mainly pain-free before that; A few occasions with pain with mainly pain-free periods in between; Slight but noticeable pain most of the time and a couple of times with severe pain; Pain that goes up and down all the time with occasions of severe pain; Pain all or almost all the time); Have you had a headache in the last 4 weeks? (No; Yes, mild; Yes, moderate; Yes, severe); Have you had pain in the jaw joints in the last 4 weeks? (No; Yes, mild; Yes, moderate; Yes, severe); Have you had pain in your arms and/or hands in the last 4 weeks? (No; Yes, mild; Yes, moderate; Yes, severe); Have you had hip pain in the last 4 weeks? (No; Yes, mild; Yes, moderate; Yes, severe); Have you had knee pain in the last 4 weeks? (No; Yes, mild; Yes, moderate; Yes, severe); Have you had pain in your ankles in the last 4 weeks? (No; Yes, mild; Yes, moderate; Yes, severe); Have you had pain in your feet in the last 4 weeks? (No; Yes, mild; Yes, moderate; Yes, severe).

3) Questions (response categories) related to use of health resources and impact of pain on work characteristics:

How often have you used over-the-counter painkillers in the last 4 weeks? (Virtually every day; Several times a week; Sometime a week; Every once in a while; Never); How often have you used prescription painkillers in the last 4 weeks? (Virtually every day; Several times a week; Sometime a week; Every once in a while; Never); Have you sought care for physical ailments in the last 12 months? (Yes, in the last 3 months; Yes, earlier in the year - but not in the last 3 months; No); Have you sought care for personal problems or mental health problems in the last 12 months? (Yes, in the last 3 months; Yes, earlier in the year - but not in the last 3 months; No); To what extent have you experienced back/neck problems affecting work performance? (Not at all; To a small degree; To a moderate degree; To a high degree; Have not worked); To what extent have you reduced working hours/changed work tasks due to back/neck problems? (Not at all; To a small degree; To a moderate degree; To a high degree; Have not worked); How many days have you been away from work due to back/neck problems? (Days: 0; < 25; > 50).
